# Role of sonication pre-treatment and cation valence in the sol-gel transition of nano-cellulose suspensions

**DOI:** 10.1038/s41598-017-11649-4

**Published:** 2017-09-11

**Authors:** C. A. Maestri, M. Abrami, S. Hazan, E. Chistè, Y. Golan, J. Rohrer, A. Bernkop-Schnürch, M. Grassi, M. Scarpa, P. Bettotti

**Affiliations:** 1 Nanoscience Laboratory, Department of Physics, University of Trento, Via Sommarive 14, 38123 Povo (TN), Italy; 2 Department of Engineering and Architecture, University of Trieste, Piazzale Europa 1, 34127 Trieste, Italy; 3 Ilse Katz Institute for Nanoscale, Science and Technology, Ben Gurion University of the Negev, Beer Sheva, 84105 Israel; 4 Department of Materials Engineering, Ben Gurion University of the Negev, Beer Sheva, 84105 Israel; 5 Department of Pharmaceutical Technology, Institute of Pharmacy, University of Innsbruck, Innrain 80/82, Innsbruck, Austria

## Abstract

Sol-gel transition of carboxylated cellulose nanocrystals has been investigated using rheology, SAXS, NMR and optical spectroscopies to unveil the distinctive roles of ultrasound treatments and addition of various cations. Besides cellulose fiber fragmentation, sonication treatment induces fast gelling of the solution. The gelation is independent of the addition of cations, while the final rheological properties are highly influenced by the type, concentration and sequence of the operations since the cations must be added prior to sonication to produce stiff gels. The gel elastic modulus was found to increase proportionally to the ionic charge rather than the cationic size. In cases where ions were added after sonication, SAXS analysis of the Na^+^ hydrogel and Ca^2+^ hydrogel indicated the presence of structurally ordered domains in which water is confined, and 1H-NMR investigation showed the dynamics of water exchange within the hydrogels. Conversely, separated phases containing essentially free water were characteristic of the hydrogels obtained by sonication after Ca^2+^ addition, confirming that this ion induces irreversible fiber aggregation. The rheological properties of the hydrogels depend on the duration of the ultrasound treatments, enabling the design of programmed materials with tailored energy dissipation response.

## Introduction

Nanocellulose (NC) is a renewable and biocompatible material with interesting and versatile properties which allow its integration in a huge number of applications, as has been extensively reviewed^1, 2^. The procedures to break natural cellulose and obtain nano-sized structures are usually based on the combination of chemical modification or enzymatic hydrolysis with mechanical refinement^3, 4^. Fine changes of these procedures give rise to different nanostructure morphology: branched nanofibrils with amorphous regions and rod-like rigid nanocrystals^5^. TEMPO-mediated oxidation of cellulose followed by sonication provides well dispersed, negatively charged cellulose nanocrystals (hereafter, TOCs)^6^.

Despite the large interest on NC and its applications, several basic aspects regulating NC properties and its interaction with the environment are still unclear. Concerning the structure investigation, effort has been addressed mainly tounderstandthe liquid crystalline self-assembly resulting in ordered helical structures with peculiar mechanical and optical properties^7–12^. The self-assembly of NC or NC-composites into soft hydrogels^13, 14^ has been characterized in terms of macroscopic parameters such as mesh size, charge density, gelation rate, mechanical performances, or stability^15–18^. In this context, rheology experiments have been performed and a gel-like behavior of NC suspensions with an elastic response even at a low concentration^19^ has been reported. In general, the rheological behaviour of NC suspensions is strongly dependent on NC production: mechanical fibrillation without chemical modification produces suspensions with flocculated structure, while NC which underwent chemical processes produces suspensions with better colloidal stability^20^. The static and dynamic rheological behavior of rod-like TOCs hydrogels suggests that liquid crystal domains consisting of self-organized ordered structures are present in TOCs^19^. Divalent or trivalent cations (Ca^2+^, Zn^2+^, Cu^2+^, Al^3+^, and Fe^3+^) induce gelation of negatively charged TOCs and form interconnected porous nanofibril networks^21^. Dynamic viscoelastic measurements performed on these gels and SEM images measured on dried samples^22^ reveal storage moduli and mesh sizes strongly related to the valence of the metal cations and their binding strength with carboxylate groups of TOCs. Though these results shed light on the role of the cross-linking reactions and electrostatic interactions in the formation of three-dimensional NC networks, little is known about the contribution of hydrogen bonds. For example, only recently a mechanism relating the role of covalent and hydrogen bonds to explain cellulose elastic properties has been proposed^23^ and the exploitation of recoverable physical bonds as sacrificial bonds for energy dissipation to reduce internal hydrogel damage under stress and increase fatigue resistance has been suggested^24^. Hydrogels contain huge amounts of water, which is an excellent competitor for intra-and inter- fibril hydrogen bonds, and polysaccharides are the biomolecules of excellence for the formation of hydrogen bonds. In this regard NC behaves as a typical polysaccharide and its structure and dynamics in solution strongly depends on electrostatic bonds and on the surface available for their occurrence. Different dynamic regimes of the water molecules have been observed within and on the surface of polysaccharide-based or synthetic hydrogels: free interstitial water which does not take part in hydrogen bonds with hydrogel molecules; bound water, which is directly bound to the chains and semi-bound water, with intermediate properties^25, 26^. As far as we know, the presence and the dynamic behaviour of these water molecules has not been investigated in NC hydrogels, which could substantially differ from more traditional hydrogels because they are formed by the assembly of rigid nanostructures rather than polymer chains.

In this work the mechanical and chemical sol-gel transition of TOCs have been investigated by the combined use of spectroscopic and rheological techniques. We found that rod-like TOCs undergo a sol-gel transition process apparently similar to that observed for flexible polymer chains and they form stable hydrogels containing rigid structural domains inside which water molecules are confined. Moreover the stability of the hydrogel depends on the sonication treatment and this fact foresees the possibility of fabricating programmable gels that behave differently depending on the amount of energy they have to dissipate.

## Results

### Sol-gel transition in TEMPO oxidized cellulose nanocrystals aqueous solutions

**Figure 1:**
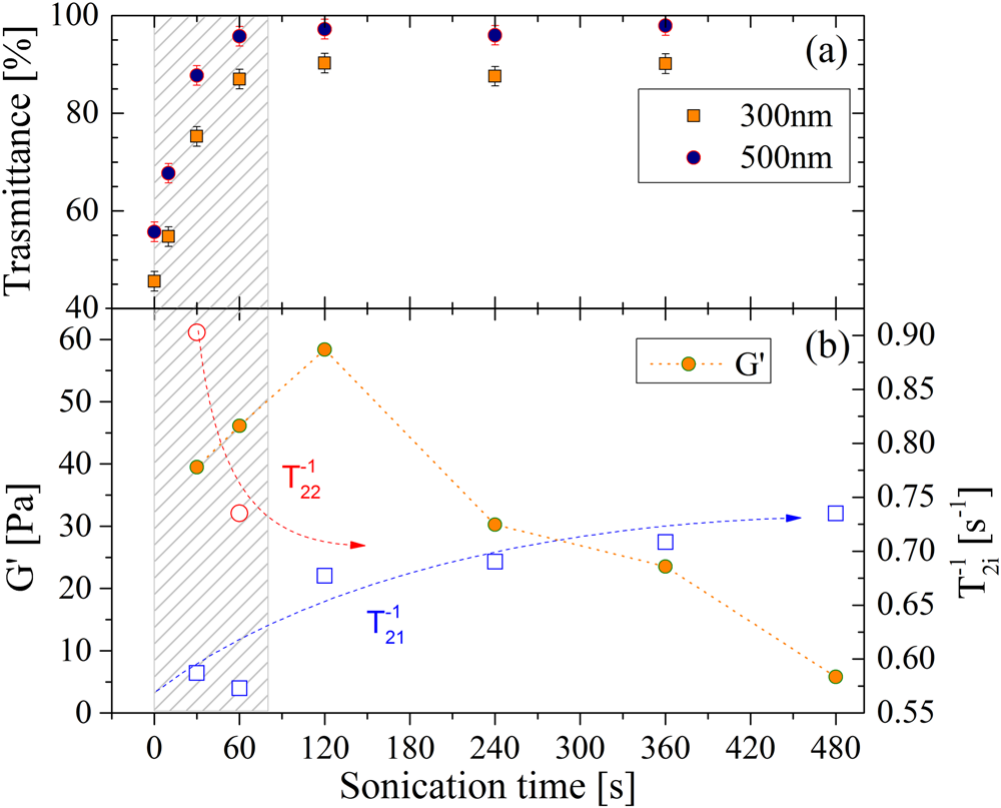
(**a**) Transmittance of TOCs vs. sonication time. Square orange points refer to UV wavelength (300 nm), while blue round points indicate visible (500 nm) wavelength. (**b**) Evolution of both elastic modulus (filled circles, left axis) and of relaxation rates (open symbols, right axis). Arrows are guides for the eyes. The shaded region underlines the sonication times during which gel structure forms.

40 mL of aqueous slurry containing 6 mgmL^−1^ TOCs at pH 7 was sonicated for variable times. Before sonication the solution was highly heterogeneous and formed by macroscopic aggregates of fibers. The turbid and flocculent suspension became progressively a homogeneous and viscous jelly solution with the sonication. After 120 s of sonication, the gel was uniform and transparent (as visible in Supplementary Fig. S1, where a TOCs solution before and after a 240 s sonication treatment is shown). Accordingly, the transmittance % of the TOCs suspensions reached a plateau value in the range 120–240 sonication time (see Fig. 1(a)). Investigation of morphology changes of the TOCs with sonication showed that the macroscopic aggregates of cellulose fibers (clearly visible with optical microscopy as shown in Supplementary Fig. S2(a) for TOCs after 30 s sonication) turned into rod-like nanocrystallites (as shown in Supplementary Fig. S2(b) after 480 s sonication). Complete disentanglement and fragmentation of the macroscopic fibers is achieved after 120 s sonication time. The quantitative transformation of cellulose fibers into TOCs after this sonication time is confirmed by the optical transmittance measurements: the average transmission value of 97% at 500 nm, rules out the presence of both macro-fibers and TOCs aggregates, as they would have induced scattering. The quantitative dispersion of the TOCs is driven by their polyelectrolyte nature. In fact at pH 7 the TOCs bear an average negative charge on the 29% of the cellobiose units, as determined by conductometric titrations (as reported in section “Methods”).

The dynamic rheological behaviour of 6 mg/ml TOCs suspensions obtained by frequency sweep tests confirms the results of the optical properties and of the structural evolution of the fibres. Indeed, frequency sweep tests indicate a gel-like behaviour of the samples sonicated for 30–360 sec, being G′ > G″, and both G′ and G″ roughly independent from the frequency. Figure 1(b) presents the dependence of G′ (conventionally evaluated at 1 Hz) versus sonication time. It shows that gel strength (i.e. its elastic component) increases up to 120 s and then decreases, suggesting a reduction of the interaction points (crosslinks) among different TOCs rods. However, the typical gel behaviour is always attained whatever the sonication time.

Transversal nuclear magnetic relaxation rate (T_2_^−1^) of water is used to investigate the state and dynamics of water in the TOCs suspensions and hydrogels at different sonication times. Multi-exponential analysis of the magnetization decay reveals that, for short sonication times (shaded region in Fig. 1(a), for sonication time up to 120 s), two relaxation times (T_21_ and T_22_) are required to describe relaxation of the magnetization. This fact suggests the coexistence of two different proton environments: macroscopic dispersed fibres correlate with the slower relaxation rate (more similar to that of free water molecules), while homogeneously, randomly arranged nanocrystals are compatible with the faster rates. Once the gel is completely formed, a single time component is sufficient to describe the system, thus supporting the idea of a homogeneous structure of the material. Moreover the average relaxation time (T_2m_) decreases with the sonication time, indicating that fibers progressively detach from each other so that the polymeric surface available for the interaction with water molecules increases (data about T_2m_ are reported in Supplementary Fig. S3).

Despite their gel nature, the connectivity within these gels is not very high, probably because of the high rigidity and limited length of TOCs, which forbid the formation of highly entangled structures. In fact, the flow curves show that, for sonication times lying in the range 30–360 s, a sudden drop of viscosity happens at stress around 20–60 Pa (see Supplementary Fig. S4) that indicates a fracture of the internal structure. Indeed, assuming that the shear modulus of the gels is represented by the average *G*′ value over the frequency range explored and that Flory theory holds^27^, the resulting average mesh size is wide (≈50–70 nm) (see section “Methods”). This datum indicates the presence of a tenuous, transient network (statistical network), similar to that observed for weak polysaccharide gels^28, 29^. Despite these considerations, the effect of sonication is not negligible as, non-sonicated TOCs suspension showed *G*′ < *G*″, a frequency dependent *G*′, and low and constant viscosity over the whole interval of stress values.

The gel shows a reversible behavior and, upon long sonication time (>360 s), it tends to revert to a viscous solution as reported by the rheological characterization (G′ as well as viscosity decrease).

The network structure formed by the TOCs was investigated by SAXS and the typical profile of aqueous solutions was found, irrespectively of the sonication time.

The overview of the experimental behavior of the aqueous suspensions of TOCs indicates that 30–120 s sonication breaks the fibrils in correspondence of the amorphous regions producing isolated TOCs which form a weak physical gel. By further sonication a solution state is reached, probably because the supplied energy breaks the hydrogen bridges between the TOCs. The role of hydrogen bonds is supported also by the dynamics of water exchange in TOCs suspensions. At 30s sonication time water is in fast exchange between the TOCs surface and the bulk, being the bulk contribution predominant since the ${T}_{21}^{-1}$ is similar to that of water. Sonication lasting 30–120 s confines the water in either the TOCs domains (with mesh size in the order of 50 nm) or in its “bulk” phase. As a consequence, the exchange rate between these two environments is slow. Further sonication uniformly disperses the TOCs in the bulk and makes more binding sites available for hydrogen bonds with water itself. In this case fast exchange regime between free and bound water holds, the transversal magnetization decay is fitted to a single exponential and the correlation time of bound water determines the increase of the measured relaxation rate.

### Sol-gel transition in salt-added TOCs solutions

Similarly to other polyelectrolyte systems (e.g. alginates^30^), cations increase the interactions among elementary elements that form the gel and drastically modify the gel properties. Despite TOCs hydrogels are not formed by long, intertwined, polymeric chains, they share several typical characteristics of these systems and cations easily induce gelation in TOC solutions. In our case sonication to disperse TOCs, followed by salt addition, produces homogeneous hydrogels; conversely, sonication performed on TOCs solutions containing 100 mM of multivalent cations produces a visually inhomogeneous hydrogel where compact macroscopic structures are surrounded by a waterish suspension (detailed flow curves for these cases are reported in Supplementary Fig. S5, while images showing the visual aspect of TOCs solutions/hydrogels in the different cases are reported in Supplementary Fig. S6).

**Figure 2:**
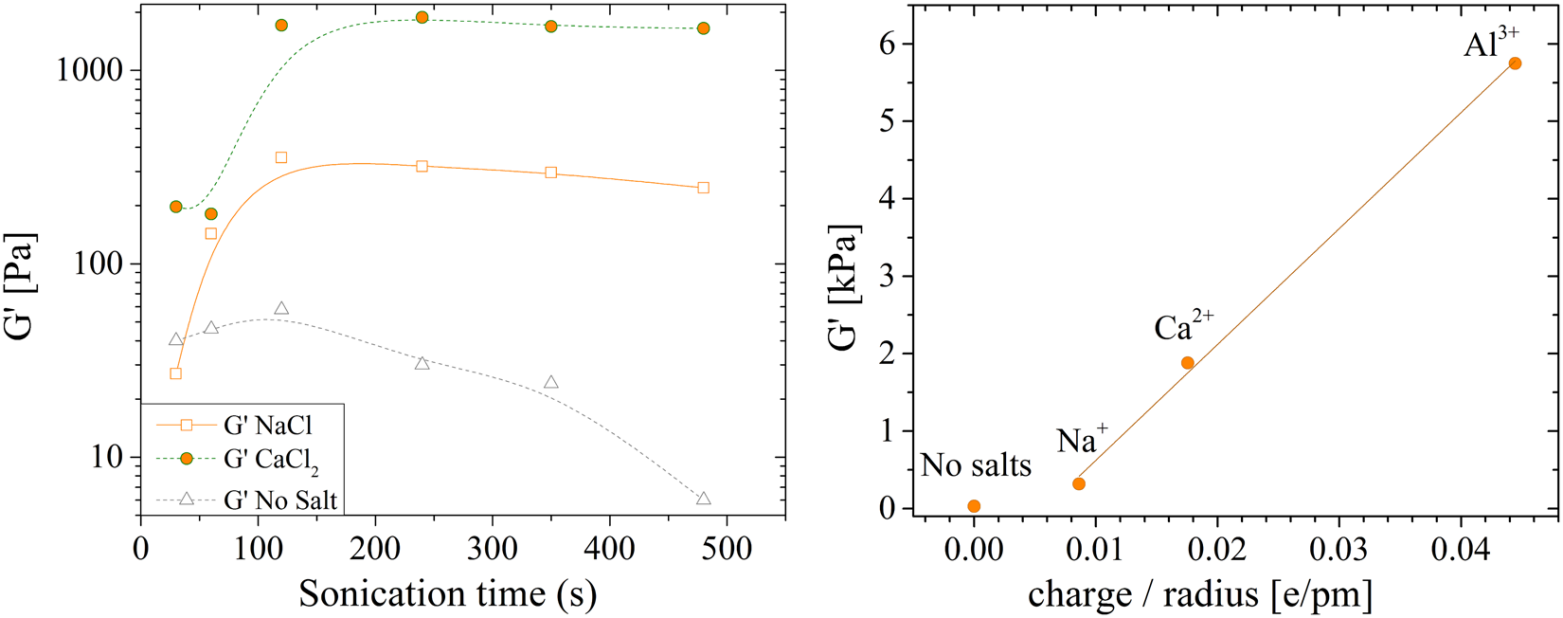
(**a**) Storage modulus measured @ 1 Hz for gels formed using 100 mM of different cations versus sonication time. Lines are guides for the eyes. (**b**) G′ vs the ratio of cation valence over cation radius for samples sonicated 240 s. The red line is the linear fit of the data.

Mechanical properties increase proportionally to cations valence and to their concentration. Figure 2(a) reports how G′ (conventionally evaluated at 1 Hz) changes upon gelation for 100 mM solutions of different cations. We found that pre-treatment of at least 120 s is required to form a homogeneous gel with a nearly stabilized G′ value.

Gel stiffness increases proportionally to cation valence. Figure 2(b) reports the value of G′ for solutions sonicated 240 s versus the ratio of cation valence over cations radius (assuming 116 pm Na^+^, 114 pm for Ca^2+^ and 67.5 pm for Al^3+^). G′ is found to increase linearly (χ^2^ = 0.999) for all the samples added with salts. We did not check higher valences because of the marked different reactivity of such cations that might introduce significant differences in the gelation mechanism. The linear trend is typical also of polymeric systems^31^ and the main difference is the reduced G′ modulus achievable with TOC nanofibers, due to their limited capability to entangle.

To note that the small G′ value obtained using Na^+^ permits the formation of a homogeneous gel irrespectively of the order of sonication and salt addition operation (as visible also in Supplementary Fig. S6(a) and (d)). This suggests that the coordination of Na^+^ by TEMPO oxidized cellulose is not strong enough to contrast the breaking of sacrificial bonds induced by ultrasonication.

Assuming that the system shear modulus G is the average *G*′ value over the frequency range explored and that Flory theory holds^27^, we estimate system average mesh size (ξ) (see section “Methods”). By this calculation (and on samples sonicated for 240 s) we obtained values of about 60, 25, 15 and 10 nm for no salt addition, 100 mM NaCl addition, 100 mM CaCl_2_ addition and 100 mM AlCl_3_ addition, respectively. As expected, mesh size is inversely proportional to G′ module of the gels.

We noticed that upon gelation (that is for sonication times longer than 120 s), ${T}_{21}^{-1}$ component assumes comparable value for both NaCl and CaCl_2_ gels and it remains constant irrespectively of the sonication time. ${T}_{22}^{-1}$ is also constant vs sonication time but it assumes different values for the gels produced with either the mono- and bi-valent cations. The suspension with no added salts shows only one component. These data are summarized in Table 1. Thus the value of ${T}_{22}^{-1}$ component is correlated with the G′ modulus of the gel (as shown also in Supplementary Fig. S7) and indicates a strong dependence of the elastic properties of the hydrogel on its local structure, mediated by both the presence of cations as well as hydrogen interactions.

**Table 1:** Water relaxation rates for pristine gels and for gels added with mono- and bi-valent salts.

Salt	${{\boldsymbol{T}}}_{{\bf{21}}}^{{\boldsymbol{-}}{\bf{1}}}$ (s^−1^)	${{\boldsymbol{T}}}_{{\bf{22}}}^{{\boldsymbol{-}}{\bf{1}}}$ (s^−1^)
NaCl^(a)^	0.57 ± 0.03	0.88 ± 0.13
CaCl_2_^(b)^	0.59 ± 0.06	1.13 ± 0.06
No Salt	0.69 ± 0.03	—

^(a,b)^100 mM salt was added to suspensions of 6 mgmL^−1^ TOCs sonicated for 240 s.

**Figure 3:**
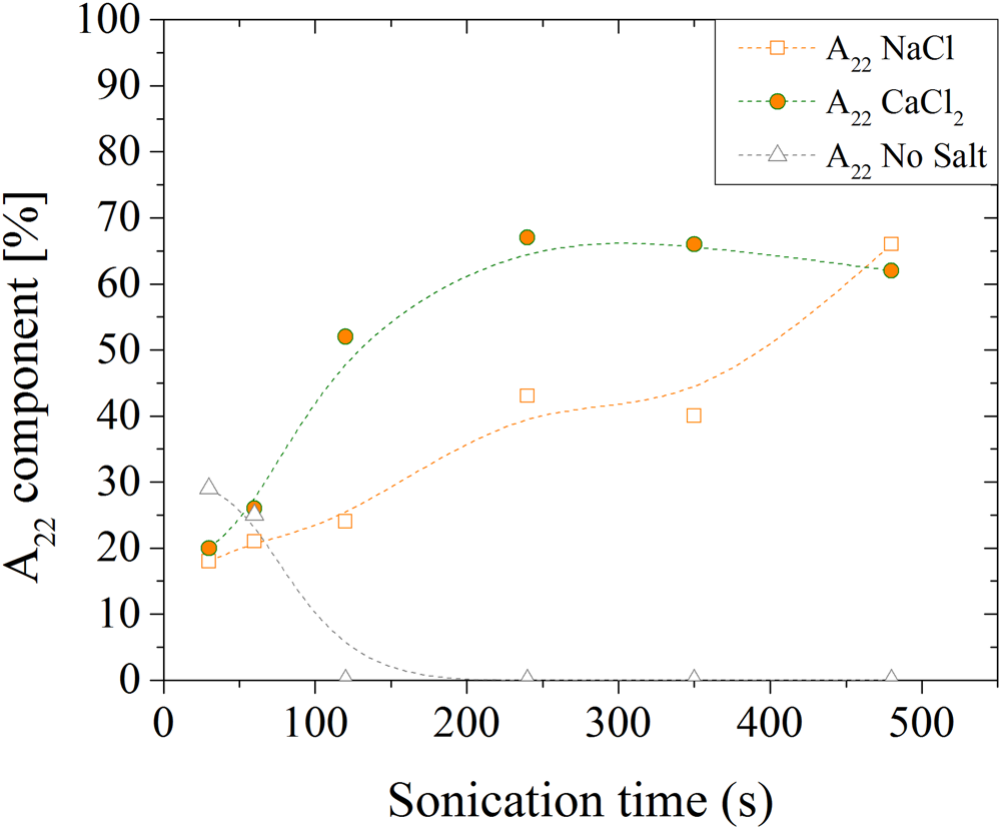
Amplitudes (A_22_) of the water magnetization components relaxing with T22 time, plotted for NaCl (squares), CaCl_2_ (circles) and solution without added salts (triangles).

Since ${T}_{22}^{-1}$ components do not change with sonication time, we are allowed to compare their relative amplitude as a function of the sonication treatment, as reported in Fig. 3.

The ${A}_{22}^{C{a}^{2+}}$ component saturates for sonication times longer than 120 s, suggesting that nanofibrils arrangement reaches a stable configuration. On the other hand, ${A}_{22}^{N{a}^{+}}$ steadily increases supporting the idea that the structure of the Na^+^-induced gel is weak and dependent on the initial conditions (that is the electrostatic interaction of Na^+^ is not enough to structure the gel). As pointed out previously, the ${A}_{22}^{sol}$ component of pure solutions disappear for sonication times longer than 120 s.

**Figure 4:**
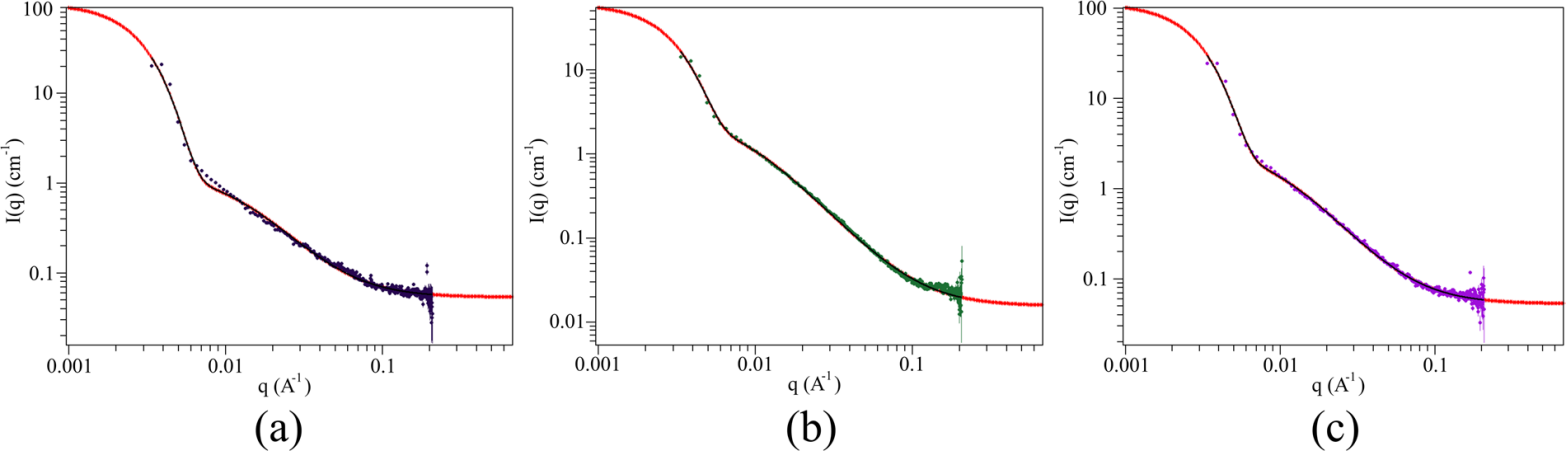
SAXS profile of sonicated TOCs suspensions after salt addition. Suspensions of 6 mgmL^−1^ TOCs have been sonicated for 240 s then 100 mM NaCl (**a**), CaCl_2_ (**b**) and AlCl_3_ (**c**) were added. The dots are the experimental points, the red line is the fit of the experimental points with the Gauss-Lorentz Gel Model (see Eq. 1 in Section “Methods”).

The hydrogels obtained after addition of NaCl, CaCl_2_ and AlCl_3_ to TOC suspensions sonicated for 240 s were investigated by SAXS measurements. Typical spectra are reported in Fig. 4.

SAXS analysis does not reveal any peak indicating the presence of structurally ordered domains. The SAXS profiles of the hydrogels (obtained adding 100 mM NaCl CaCl_2_ and AlCl_3_ to TOCs solutions sonicated for 240 s) were properly fitted with the Gauss-Lorentz models (see Eq. 4 in Section “Methods”) and point out the presence of two structural length scales, whose values are reported in Table 2. The static correlation length is attributed to the average size of long-lived entanglements; on the other hand the dynamic correlation length is associated to the fluctuation amplitudes between crosslinks^32, 33^.

**Table 2:** Static and dynamic correlation lengths of TOCs hydrogels formed by the addition of different salts (100 mM) after 240 s sonication.

Salt (100 mM)	Static length (Å)	Dynamic length (Å)	Mesh size (Å)^(d)^
NaCl	416 ± 144^(c)^	77 ± 7^(c)^	250
CaCl_2_	474 ± 28^(a)^	127 ± 11^(a)^	150
AlCl_3_	469 ± 51^(b)^	106 ± 13^(b)^	100

^(a)^average and standard deviation (s.d.) of 3 different samples; ^(b)^average and s.d. of 5 different samples; ^(**c)**^average and s.d. of 4 different samples; ^(d)^values estimated assuming Flory theory.

Notably, both static and dynamic correlation lengths are independent of the crosslinking cation and their values do not easily correlate with the characteristic nanocrystal size (about 300 × 5 × 5 nm). The static correlation length might describe the typical size of high density regions formed by the overlapping TOCs. Under these assumptions, the density of TOCswould be constant across all samples (as confirmed by both optical and NMR analysis), irrespectively of the cations. On the other hand, the dynamic correlation length depends on the local gel structure as it is affected by the strength of the crosslink created by the different cations. Thus, it directly correlates with the G′ of the gel. The last column of Table 2 reports the values of the mesh sizes as estimated from the Flory theory. While there is a large discrepancy between SAXS and Flory theory predictions for gels formed using NaCl, the values reasonably agree for gels formed using multivalent cations.

On the contrary of findings reported by other authors on similar gels^34^, our samples do not show any ordered domain and we found that stiffer materials have structure made by small meshes. These differences are probably due to the thoroughly washing of the sample we use to neutralize the solution, instead of adding HCl. Our neutralization procedure avoids the uncontrolled increase of salt content and of the ionic strength of the solution and reflects in a material where water-mediated hydrogen bonds networks dominate in the absence of added salts. This fact determines different material properties (e.g. we noticed a highly increased gas barrier property of TOCs films obtained with this method^35^).

**Figure 5:**
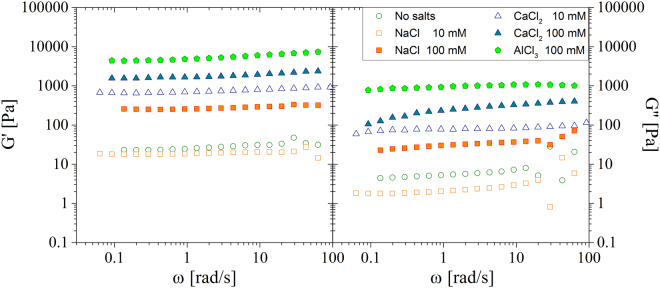
Frequency sweep tests of TOCs gels created adding different cations and concentrations to suspensions of 6 mgmL^−1^ TOCs sonicated for 240 s. (**a**) Elastic moduli; (**b**) viscous moduli.

The comparison of gel formed by adding 10 mM and 100 mM concentrated solutions is discussed below. Figure 5 reports the frequency sweep tests for the gels obtained using different cations.

While the cation valence has a profound influence on the gel stiffness, for a given cation, modest changes appear between the 10 mM and the 100 mM concentrations. The relatively small difference between the 10 mM and 100 mM concentrations might be due to a saturation of the TOCs sites available for crosslinks.

Finally, we investigated how the properties of the gel change with the combined role of sonication and cation addition. We found that the maximum value of G′ depends on the duration of sonication pre-treatment (done before the gelation). Depending on if and which cation is added, G′ can either increase or decrease with the sonication time. This fact suggests that gels are in metastable state. This hypothesis is confirmed by looking at the dynamics of the G′ change vs sonication time. We assume the G′ value at 120 s of sonication (${G}_{120}^{\text{'}}$) to be the highest achievable in each gel and we normalize the variation of G′ for different sonication pre-treatment against the ${G}_{120}^{\text{'}}$ value: ${\rm{\Delta }}G^{\prime} =\frac{{G}_{120}^{^{\prime} }-G^{\prime} }{{G}_{120}^{^{\prime} }}x\,100$.

ΔG′ scales inversely proportional to the valence of the crosslinking cations. That is, the pretreatment duration to bring the gel into the most stable state, scales proportionally to the cation valence. This evidence correlates with the trend of the amplitude of the T_22_ that steadily increases in NaCl systems, while saturates in CaCl_2_ for sonication times longer than 120 s. The result is also supported by the data reported in Figs 1 and 2(a): a prolonged sonication treatment reduces the density of crosslinking points and, in turn, the G′. That is, above an optimal sonication pretreatment, the material tends to liquefy again and the duration of the optimal sonication depends on the ion valence. In fact such rearrangement is hindered in stiffer gels that require longer sonication to achieve their maximum G′ and show a slower decrease (if any) of their Young’s moduli.

## Discussion

We investigated the effect of the parameters which strongly affect the assembly of TOCs in gel-like supramolecular networks: the energy released by sonication and the addition of small cations.

The overall analysis of the parameters highlights the following fundamental results:

1. TOCs which are characterized by a rod-like, rigid structure, undergo a sol-gel transition process apparently similar to that observed for flexible polymer chains. This transition can be induced by non-exhaustive sonication, even though a clearer gel-like behavior is obtained if sonication is followed by salt addition
2. the gelation mechanism is cation-dependent and mediated by hydrogen interactions
3. the stability of the gel depends on the duration of the sonication treatment.

Compared to truly polymeric systems formed by long, entangled molecular chains, short and rigid TOCs yet form gels thanks to the strong electrostatic interactions generated by hydrogen bonds and between cations and carboxyl groups. Gelation is nearly instantaneous and it starts by simply hand shaking a sonicated solution added with cations. The fact that homogeneous gels are formed only if salts are added to fibres well dispersed by sonication suggests that strong interactions take place between the cations and the non-sonicated fibres that form inhomogeneous macroscopic aggregates. These aggregates limit the efficacy of the sonication treatment, contrast the dispersion of isolated nanocrystal and hinder the formation of a homogeneous gel phase.

These hypotheses are confirmed by the different behaviour of the gels formed by adding either mono- or multi-valent cations: softer gels (NaCl) have wider meshes that reduce their G′ modulus. Moreover, despite the weaker interactions produced by Na^+^, the flow curves of these gels shows a drop of the viscosity larger than the corresponding for the inhomogeneous gel formed with Ca^2+^: the weaker interactions created by monovalent cations permit them to disperse homogeneously in the gel phase independently on the order of salt addition and sonication. On the other hand both CaCl_2_ and AlCl_3_-added gels form homogeneous phases only if cations are free to diffuse around isolated nanocrystals, that is only if TOCs are already well dispersed by the sonication. In the case of Ca^2+^, the flow curves clearly show a higher threshold value for the viscosity drop compared to the Na^+^-based gels.

**Figure 6:**
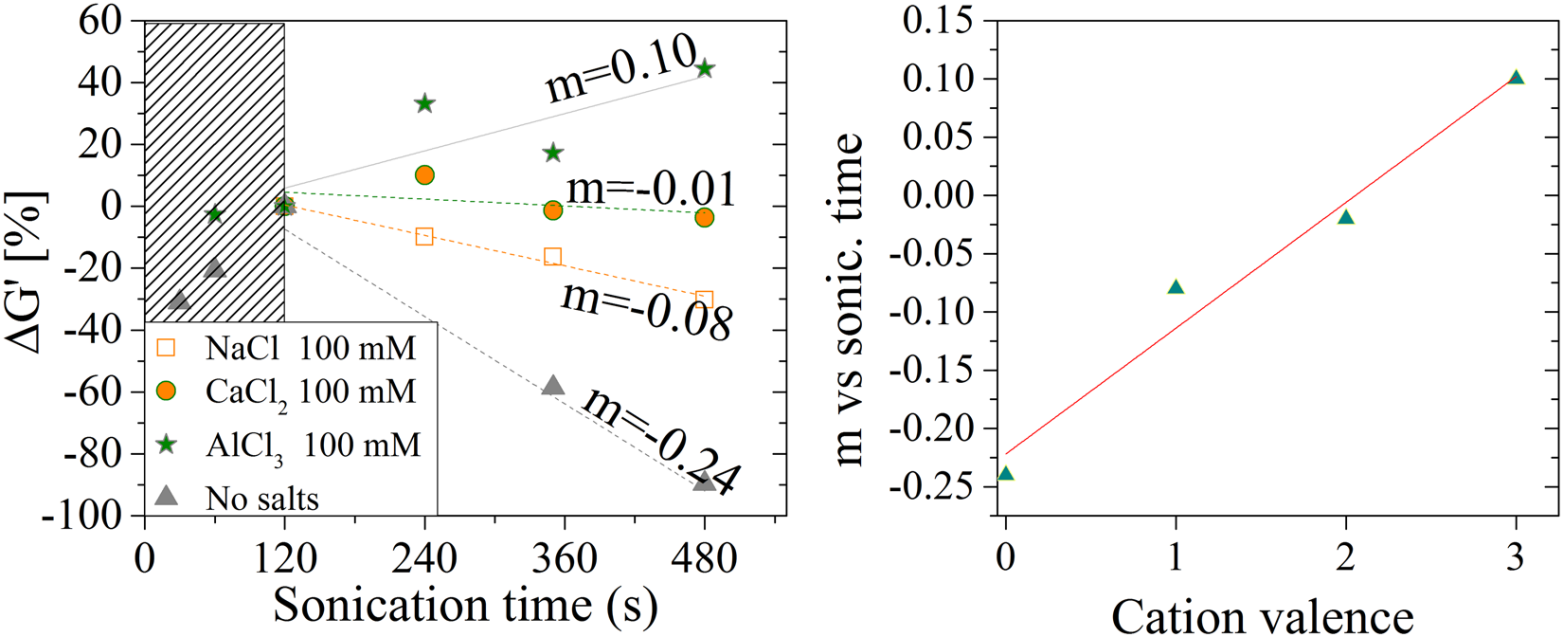
(**a**) Variation of G′ (conventionally evaluated at 1 Hz) vs sonication treatment for different cations (the data are the same reported in Fig. 2). (**b**) Plot of the slope of G′(1 Hz) variation versus cation valence.

The homogeneity of gels formed by TOCs is confirmed by both optical and NMR analysis: gel transparency is very high and essentially no scattering appears in transmittance measurements, while NMR reveals nearly constant ${T}_{21}^{-1}$ rates for all gels, while the ${T}_{22}^{-1}$ parameter scales with the G′ modulus and with the duration of the sonication treatment. SAXS analysis has unveiled that all gels contain high-density domains of about 40–50 nm. Water confined in these domains exchanges slowly with the less structured regions, giving rise to a compartmentalized relaxation behavior. These high-density domains are independent of the added cation (i.e. similar dimensions and water relaxation rate were obtained). However the elasticity of the network is cation dependent, as shown in Fig. 2, and Ca^2+^ is more effective than Na^+^ in forming such rigid domains. This fact is confirmed by the higher value of the amplitude of the magnetization component relaxing with rate ${T}_{22}^{-1}$, which is ascribable to the overall number of water molecules confined in the rigid domains. This amplitude reaches its maximum value (about 70%) for Ca^2+^ added to TOCs sonicated for more than 120 s. Conversely, if Na^+^ is added longer sonication times are required for the same amount of confined water. These results suggest that beyond the electrostatic interaction, the coordination by carboxylated groups contributes to the Ca^2+^ effect, and are in line with the interaction models of cations with nanocellulose fibres proposed by^36^ and with the stability of metal carboxylate complexes measured by^37^. Conversely, non-specific electrostatic interactions seem to predominate in the case of Na^+^, which necessitates of more extensive sonication, which breaks the hydrogen bonds between the TOCs and forms weaker gels. Unfortunately, similar conclusions cannot be extended to the case of Al^3+^ because it affects the magnetization decay. We suppose the different behaviour of the Al^3+^ to be related to a faster gelation dynamics induced by trivalent cation that produces inhomogeneous gels structures. Probably a more homogeneous dispersion of the cations across the gel volume would achieve a more stable state (stiffer gel). Such hypothesis is indirectly supported by the data reported in Fig. 6 in which Al^3+^ is the only cation who does not achieve the maximum G′ upon 120 s of sonication.

These results demonstrate that, despite their non-polymeric and rigid nature, TOCs shows sol-gel transition similarly to polymers and produce gels with good mechanical properties. Moreover the short length of the TOCs (of the order of few hundreds nm) enables a very fast sol-gel transition (few seconds) and the mechanical properties of the gels can be tuned by playing with both cation valence and sonication pre-treatment. Despite the negligible entanglement shown by the TOCs and the fast initial stage of gelation induced by multivalent cations, reaching the maximum G′ -for a given preparation condition- is a complex and rich process as it requires long sonication times. The large amount of energy required to bring the system into a stable state is probably connected to a hindered mobility of the TOCs after the initial stage of the gelation; while the decrease of the G′ by prolonged sonication treatments, might be due to reduction of crosslinking points of the nanocrystals that, by reducing their entanglement, increases the mobility of neighbour fibres and decreases the overall gel stiffness. This behaviour might be used to design hydrogels programmed to behave differently depending on the amount of energy they have to dissipate.

## Methods

### Reagents

All reagents used were from Sigma (St. Louis, MO) and were used without further purification. Never dried soft bleach pulp (Celeste90^©^) were received from SCA-Ostrand^©^ (Sweden).

### Preparation of Nanocellulose

2,2,6,6-tetramethylpiperidinyloxy (TEMPO) oxidized cellulose nanocrystals was obtained by a slightly modifying the procedure reported in ref. 38: 1 g of cellulose pulp was swollen in 100 mL water under stirring for 1 hr. Then 0.1 g NaBr,1.75 mL NaClO and 16.2 mg TEMPO were added under vigorous stirring. The pH of the solution was maintained in the interval 10.5–11.0 by addition of 1 M NaOH until it remained almost constant (around 0.75 mL NaOH were added). Then, the slurry of TOCs was carefully purified by other chemicals and brought to pH 7 by repeated washings in ultrapure water and concentrated by a rotary evaporator (Heidolph, Schwaback Germany) to obtain a final TOCs concentration of 6 mgmL^−1^.

The sonication was performed by using an ultrasonic homogenizer (HD2200 Bandelin Sonoplus, Berlin Germany) equipped with a 13 mm titanium tip. An output power W_eff_ of 160 W was delivered in 40 mL of TOCs slurry for a variable time. A detailed analysis of TOCs morphology can be found in ref. 39.

### Rheology experiments

Rheological measurements were performed by a stress controlled rotational rheometer (Haake Mars Rheometer, 379–0200 Thermo Electron GmbH, Karlsruhe, Germany) equipped by parallel plate geometry (C35/1°, ϕ = 35 mm). The gap was fixed at 0.5 mm. The linear viscoelastic region was determined by stress sweep tests (in the range 0.01–500 Pa) keeping a constant frequency at 1 Hz. Frequency sweep tests were performed in the frequency range 0.01–10 Hz at a constant shear stress of 1 Pa (within the linear viscoelastic field) and the temperature was always set to 25 ± 1 °C.

The evaluation of the average network mesh size (ξ) was performed assuming that the shear modulus of the gels (*G*) is represented by the average *G*′ value over the frequency range explored and that Flory theory holds^27^. Indeed, Flory’s theory enables the determination of the polymeric network crosslink density ρ_x_ (defined as the moles of junctions between different polymeric chains per hydrogel unit volume) according to eq. (1):

1 $${{\rm{\rho }}}_{{\rm{x}}}=G/RT$$

where R is the universal gas constant and T is absolute temperature. The link between ρ_x_ and ξ is provided by the equivalent network theory^40^. This theory, starting from the evidence that, in most cases, a detailed description of a real polymeric network is rather complicated, if not impossible, suggests replacing the real network topology by an idealized one (cubical arrangement of the meshes) sharing the same average ρ_x_. According to this theory, the empty volume associated to each crosslink is that of a sphere centered in the crosslink and characterised by a diameter equal to the average mesh size (ξ). Remembering the definition of crosslink density, it turns out that the volume competing to each cross-link in the real network (1/(N_A_ρ_x_); N_A_ = Avogradro number) equals the volume of each sphere as the two networks share the same ρ_x_. Thus, the relation between ξ and ρ_x_ reads:

2 $$\frac{{\rm{4}}}{{\rm{3}}}{\rm{\pi }}{(\frac{{\rm{\xi }}}{{\rm{2}}})}^{{\rm{3}}}=\frac{{\rm{1}}}{{{\rm{\rho }}}_{{\rm{x}}}{N}_{{\rm{A}}}}= > {\rm{\xi }}=\sqrt[3]{6/{{\rm{\pi }}{\rm{\rho }}}_{{\rm{x}}}{N}_{{\rm{A}}}}$$

Optical measurements have been performed on a Varian Cary 5000 UV-VIS-NIR spectrophotometer. TOCs samples were placed in a 1 mm optical path cuvette and scanned in the range 200–800 nm with a 1 nm resolution. As a baseline correction the signal of the empty cuvette was considered.

To note that optical and rheological analysis have been done on different samples prepared under the same conditions.

### Nuclear Magnetic Resonance (NMR) measurements

The dynamics of water in the hydrogels was investigated by low-field 1H-NMR. To this purpose, the water protons transverse relaxation time (T_2_) of the hydrogels and of the NC suspensions was measured at 25 °C by a Bruker Minispec mq20 operating at 20.1 MHz (Karlsruhe, Germany). The CPMG (Carr–Purcell–Meiboom–Gill) sequence {90°[-τ−180°-τ(echo)]_n_-*T*_R_} with a 8.36 μs wide 90°pulse, τ = 250 μs and *T*_R_ (sequences repetition rate) equal to 5 s was used. The criterion adopted to choose *n* consisted in ensuring that the final FID intensity were about 2% of the initial FID intensity (in the light of this acquisition strategy, we verified that it was un-necessary adopting *T*_R_ > 5 s). Accordingly, *n* was approximatively equal to 700. Finally, each FID decay, composed by *n* points, was repeated 36 times (number of scans). The relaxation times distribution (*A*_i_, *T*_2i_) was determined by fitting the FID time decay (*I*_*s*_), related to the extinction of the *x*–*y* component of the magnetization vector (*M*_*xy*_), according to its theoretical estimation *I*(t):

3 $$I(t)=\sum _{i=1}^{m}{A}_{i}{e}^{-\frac{t}{{T}_{2i}}}$$

where t is time, A_i_ is the amplitude of the magnetization of the protons relaxing with time T_2i_. The number of exponential m was determined by minimizing the product χ^2^*(2 m), where χ^2^ is the sum of the squared errors and 2 m represents the number of fitting parameters of Eq. (1)^41^.

Microscopy measurements: a digital Atomic Force Microscope (NT-MDT Universal SPM scanning head SMENA) equipped with a Si tip, operating in semi-contact mode was used to investigate the morphology, size and size distribution of the TOCs. TOCs solutions were suitably diluted with water to obtain isolated fibers. A drop of this solution was deposited on a silicon support and dried at 60 °C in an oven. Image analysis was performed using the software Gwyddion v2.46^42^.

### Small angle X ray scattering (SAXS)

SAXS measurements have been used to calculate the scattering from the hydrogels. We used the Gauss-Lorentz Gel Model that is assumed to be valid for gel structures^43^ that modeled the scattered intensity (Iq) as a sum of a low-q exponential decay plus a lorentzian at higher q-values:

4 $$I(q)={I}_{G}(0)\exp (-\frac{{q}^{2}{{\rm{\Xi }}}^{2}}{2})+\frac{{I}_{L}(0)}{(1+{q}^{2}{\xi }^{2})}$$

where ${\rm{\Xi }}$ is the static correlation length in the gel, which can be attributed to the ‘frozen-in’ crosslinks of some gels, ξ is the dynamic correlation length, related to the fluctuating polymer chain between crosslinks. I_G_(0) and I_L_(0) are the scaling factors for each of these structures.

### Conductometric titrations

The pH of a TOCs suspension (2 mgml^−1^) was brought to 2.0 by HCl and 1 mM NaCl was added. Then, the pH was raised until 13.0 by addition of small aliquots of sodium hydroxide (0.1 M). The pH and conductivity values were recorded after each addition by a HD 2256.2 (Delta Ohm (Padova, Italy) instrument.

All data generated or analysed during this study are included in this published article (and its Supplementary Information files).

## Electronic supplementary material

Supplementary information
