# Supplementary material for: Role of sonication pre-treatment and cation valence in the sol-gel transition of nano-cellulose suspensions
Source: Sci Rep. 2017 Sep 11;7:11129. doi: 10.1038/s41598-017-11649-4 (PMC5593908; doi:10.1038/s41598-017-11649-4)
Supplement: Supplementary file 1 — Supplementary information [file 41598_2017_11649_MOESM1_ESM.pdf]

Supplementary information

Role of sonication pre-treatment and cation valence in nano-cellulose suspensions sol-gel transition.

C. A. Maestri, M. Abrami, S. Hazan, E. Chistè, Y. Golan, J. Rohrer, A. Bernkop-Schnürch, M. Grassi, M. Scarpa, P. Bettotti

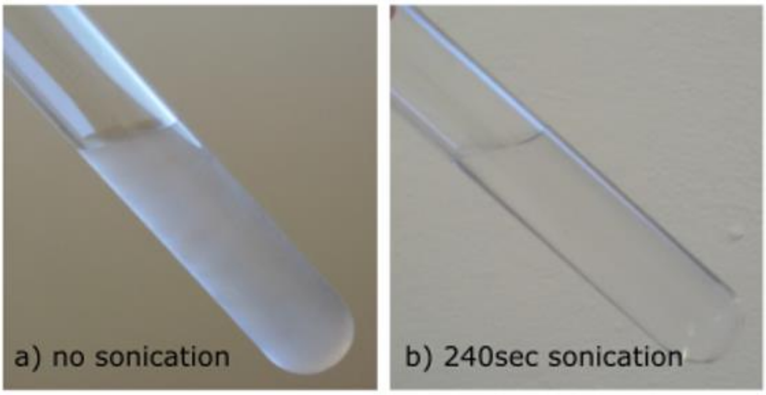

Fig S1. Visual appearance of (a) non sonicated TOCs slurry and (b) sonicated one.
Macroscopic aggregates of cellulose fibers induces the whitish appearance of the non-sonicated slurry. Upon sonication, fibers are disrupted and transformed in TOCs leading to transparent, homogeneous solution.

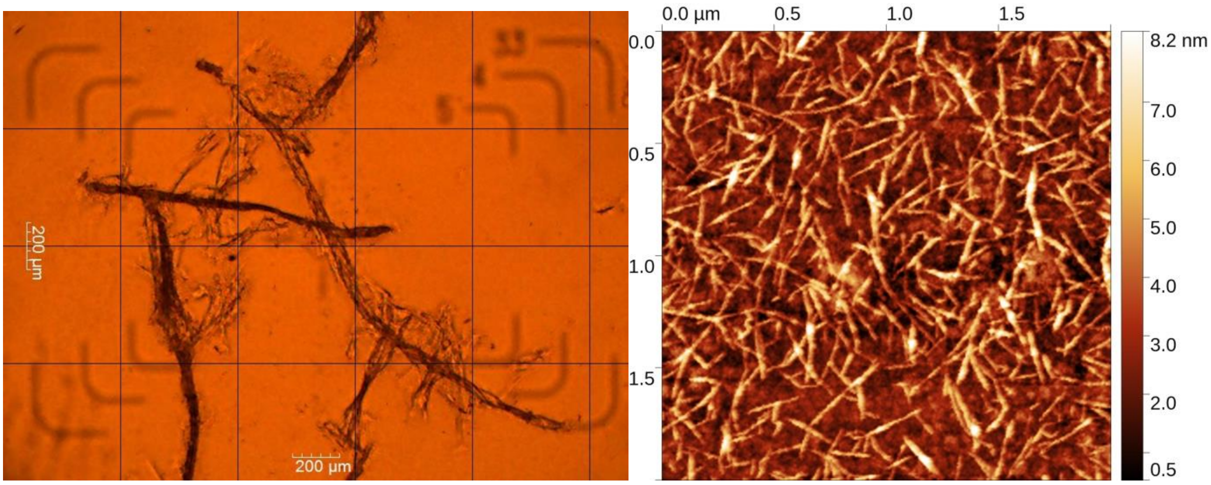

(a) (b)

Figure S2. (a) Optical microscopy image of macroscopic cellulose fibers after a short sonication treatment (30 s); (b) Atomic force microscopy of cellulose nanocrystals after a prolonged sonication treatment (480 s).

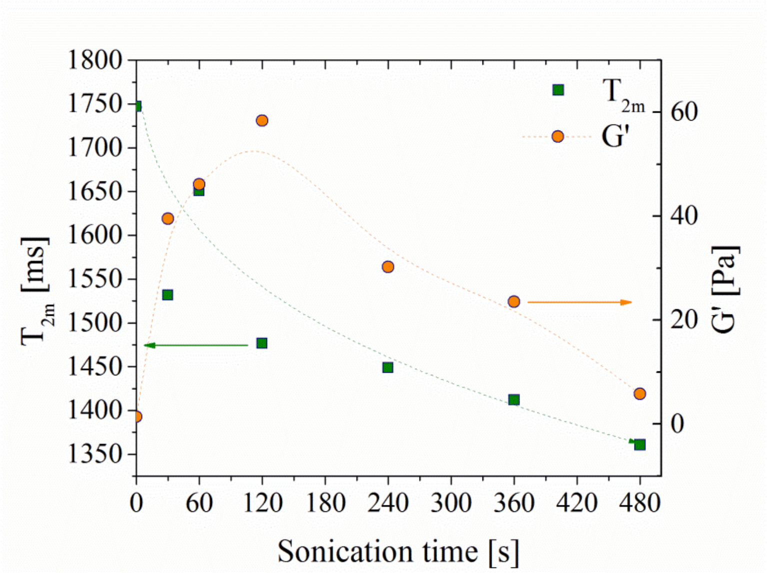

Figure S3. (Left axis) T_{2m} data for samples without salt addition. A single decay time describes the magnetic relaxation. (Right axis) The G' modulus corresponding to different sonication times. The decrease of G' vs sonication time support the hypothesis that a more homogeneous dispersion of the fibres is less entangled and exposes a larger surface available to interact with water molecules (lines are guides for the eyes).

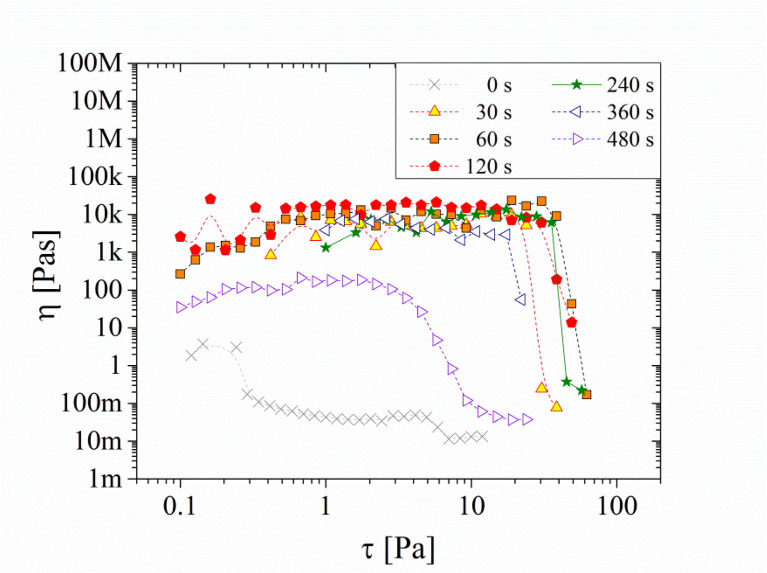

Figure S4. Flow curves for gels without salts. The system shows a dependence of its viscosity vs. sonication time. The maximum value of η is obtained for sonication time between 30 and 240 s. In all cases η shows a marked drop for shear stress above 30 Pa

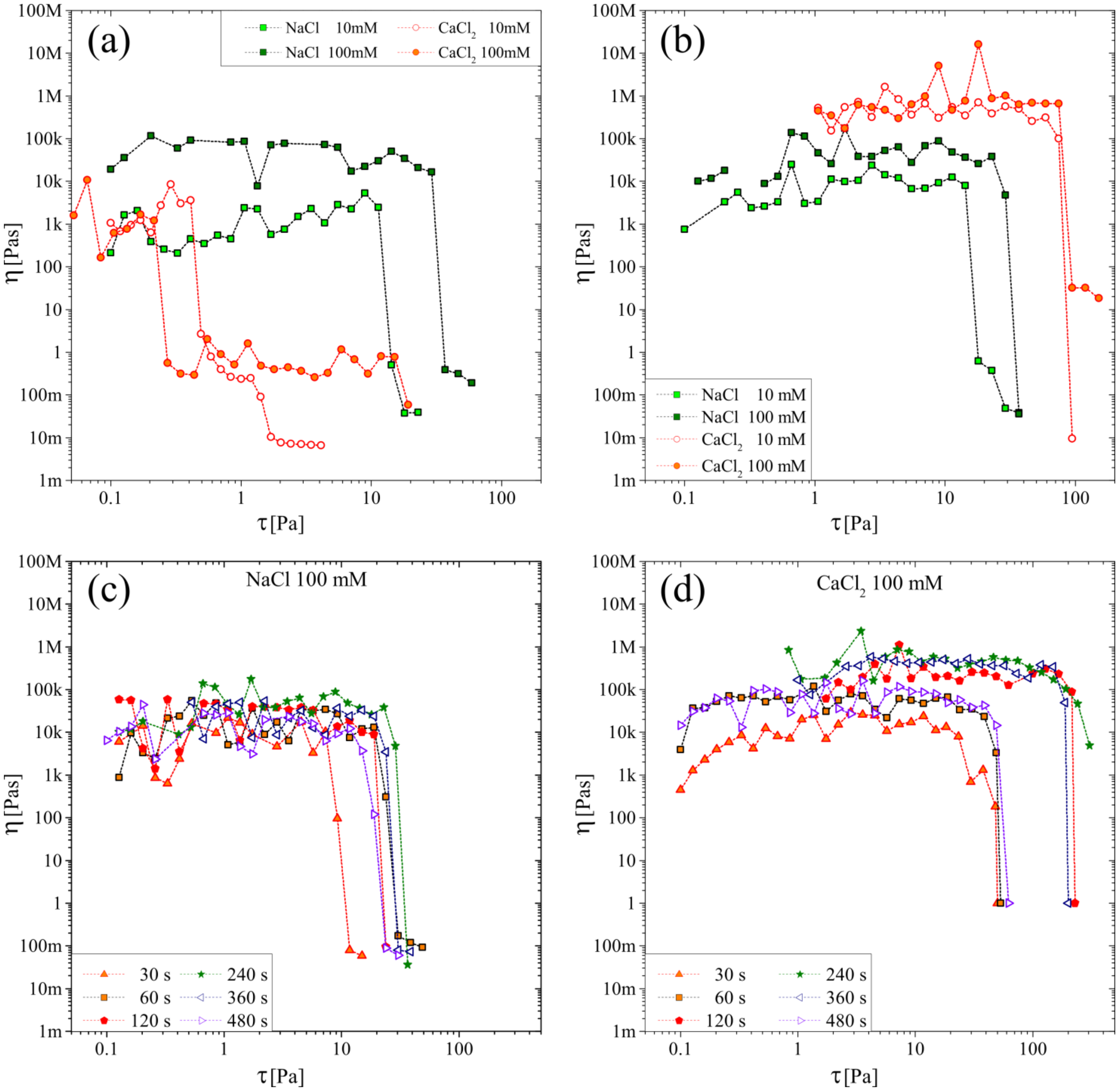

Figure S5. Flow curves for gels formed upon salts addition. (a) Addition of salts followed by sonication of the TOCs creates an inhomogeneous gel. The Na-based gels are homogeneous, compared to the Ca-based systems, and this fact reflects into a larger value of the viscosity drop, even if they show a smaller G' modulus. (b) If salts are added after sonication, homogeneous gel are formed and flow curves show a clear increase of the inflection point versus cation valence and concentration. (c) Gels formed by addition of 100 mM NaCl after sonication show an increase of threshold shear stress that determines the decrease of the viscosity for sonication times up to 240 s, then the threshold is slightly reduced for longer sonication treatment. (d) In the case of 100 mM $CaCl_2$, added after sonication, the threshold shear stress is, again, maximized for 240 s long sonication, but its decrease is more marked compared to NaCl case.

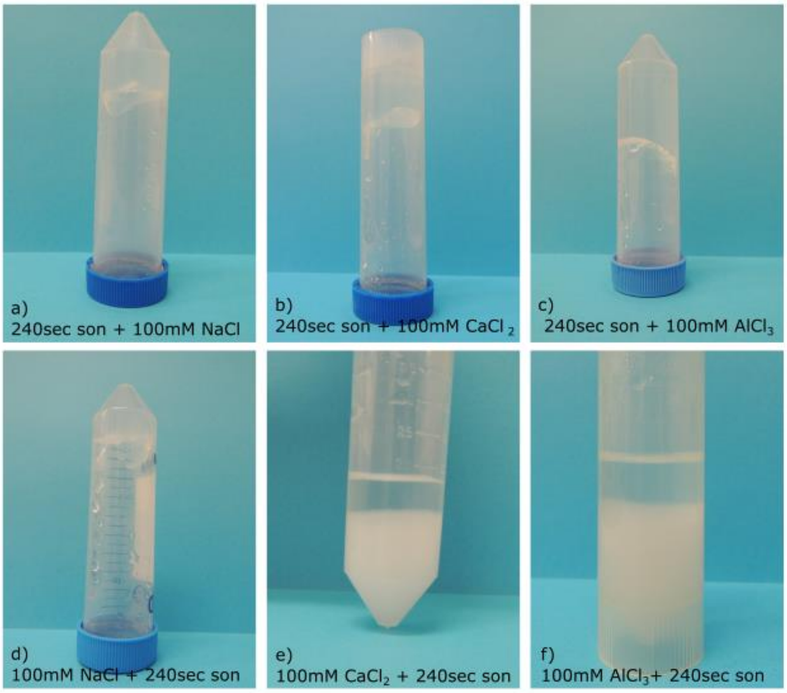

Figure S6. Visual appearance of TOCs solution/hydrogels. (a,b,c) homogeneous and transparent gels formed by 240 s sonication treatment followed by cation addition. (d,e,f) samples obtained by first adding the cations before the sonication. Only NaCl forms a weak and homogeneous gel, while solutions containing multivalent cations form inhomogeneous materials. Probably cations induce a gelation of the outer surface of the macroscopic fibers aggregates that prevents an effective sonication and dispersion of TOCs.

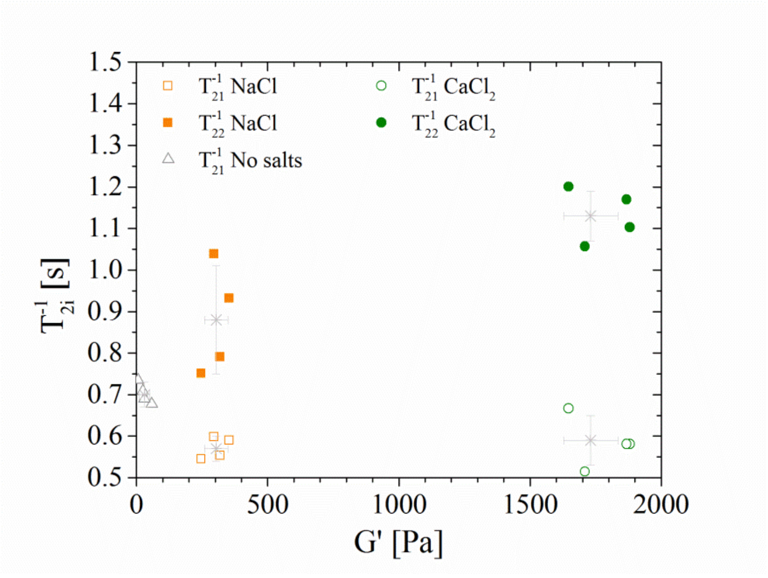

Figure S7. Correlation between T_{2i} components and G' for both gels formed with and without salts. Only data for samples added with 100 mM salts and sonicated for at least 120 s are shown. Assuming the T_{2i} components describes the local environment of the water molecules, the gel formed without salt addition shows a T_{21} relaxation rate intermediate compared to the two components needed to describe the magnetization relaxation in gels formed upon salt addition. Cation induced gels show a comparable T_{21} rate for both NaCl and $CaCl_2$ samples, while the T_{22} rate is significantly larger in $CaCl_2$ containing gels, indicating a different local structure of the water molecules that determines the greater G' modulus achieved in these gels.
